# Cardiovascular disease and mortality following placental abruption

**DOI:** 10.1093/aje/kwaf289

**Published:** 2026-01-20

**Authors:** Cande V Ananth, Rachel Lee, Linda Valeri, Sonia M Grandi, Todd Rosen, William J Kostis, for the PACER Study Investigators

**Affiliations:** Division of Epidemiology and Biostatistics, Department of Obstetrics, Gynecology, and Reproductive Sciences, Rutgers Robert Wood Johnson Medical School, New Brunswick, NJ, United States; Rutgers Robert Wood Johnson Medical School, Cardiovascular Institute of New Jersey, New Brunswick, NJ, United States; Department of Medicine, Rutgers Robert Wood Johnson Medical School, New Brunswick, NJ, United States; Department of Biostatistics and Epidemiology, Rutgers School of Public Health, Piscataway, NJ, United States; Rutgers Robert Wood Johnson Medical School, Environmental and Occupational Health Sciences Institute, Piscataway, NJ, United States; Division of Epidemiology and Biostatistics, Department of Obstetrics, Gynecology, and Reproductive Sciences, Rutgers Robert Wood Johnson Medical School, New Brunswick, NJ, United States; Department of Biostatistics, Joseph L. Mailman School of Public Health, Columbia University, New York, NY, United States; Department of Epidemiology, Harvard T.H. Chan School of Public Health, Boston, MA, United States; Child Health Evaluative Sciences, The Hospital for Sick Children, Toronto, ON, Canada; Division of Epidemiology, Dalla Lana School of Public Health, University of Toronto, Toronto, ON, Canada; Division of Maternal-Fetal Medicine, Department of Obstetrics, Gynecology, and Reproductive Sciences, Rutgers Robert Wood Johnson Medical School, New Brunswick, NJ, United States; Rutgers Robert Wood Johnson Medical School, Cardiovascular Institute of New Jersey, New Brunswick, NJ, United States; Department of Medicine, Rutgers Robert Wood Johnson Medical School, New Brunswick, NJ, United States

**Keywords:** placental abruption, cardiovascular disease, heart disease, stroke, mortality, multistate models, unmeasured confounding

## Abstract

We investigate the relationship between placental abruption and cardiovascular disease (CVD) events using a population-based, retrospective cohort study of individuals who delivered a singleton birth between 1993 and 2020 in New Jersey, USA. We fit multistate weighted Cox models to estimate the risks of non-fatal CVD hospitalization, all-cause mortality, and non-fatal CVD hospitalization to all-cause mortality. We examine these associations in two non-overlapping cohorts of individuals with their first delivery only (parity 1) and those with the first two consecutive deliveries (parity 1-2). Associations were corrected for unmeasured confounding bias. Of 2 874 671 deliveries, 1.0% (*n* = 28 913) had an abruption. The median follow-up was 16 years (range, 0-28 years). Compared to no abruption, placental abruption among first deliveries was associated with adjusted hazard ratio (HR) of 1.27 (95% confidence interval [CI], 1.11-1.46) for the transition from delivery to non-fatal CVD hospitalization; HR, 1.77 (95% CI, 1.28-2.44) for all-cause mortality; and HR, 1.52 (95% CI, 0.98-2.37) for non-fatal CVD hospitalization to all-cause mortality. The corresponding HRs for recurrent placental abruption (parity 1-2 cohort) were stronger, although less precise. Corrections for unmeasured confounding slightly attenuated these risks. These findings underscore the importance of placental abruption as a potential risk factor for maternal CVD risks.

## Introduction

Globally, cardiovascular disease (CVD) remains the leading cause of mortality, implicated in almost 20 million deaths, translating to a third of all deaths in 2022.^1^ It is now recognized that pregnancies with an obstetrical complication unmask short- and long-term CVD risks.^2^^,^^3^ One such complication is placental abruption, the premature separation of the placenta from the uterine wall, which may be caused by acute stimuli at the maternal-fetal interface, resulting in the rupture of decidual arterioles. Abruption complicates about 1% of pregnancies^4-6^ but recurs in over 10% of second pregnancies with prior abruption. This complication also portends substantial risks to the mother and perinatal risks, as well as maternal CVD complications along the life course.^7-10^ A meta-analysis reported individuals who suffered abruption were at a 2.7-fold higher risk of CVD mortality and a 1.3-fold higher risk of non-fatal CVD complications.^11^

Preeclampsia, placental abruption, and fetal growth restriction form the spectrum of ischemic placental disease.^12-14^ Risk factors that are common to all three conditions include dyslipidemia, metabolic syndrome, vascular-mediated conditions, obesity, physical inactivity, tobacco use, and alcohol.^15^ These risk factors predispose individuals to inflammation, endothelial cell dysfunction, and increased oxidative stress, which, in turn, affect the risk of abruption.^16^ These clinical and behavioral risk factors are among the most strongly implicated in CVD.^17^

We explore the extent to which placental abruption affects maternal CVD risks. Three innovative aspects underlie this study. First, the degree to which abruption contributes to mortality risk with and without an intervening CVD hospitalization remains unknown. It is also unknown whether the risk varies based on the presence of an intervening (competing) event. Previous studies have typically overlooked non-fatal events when estimating the effect of abruption on CVD mortality,^7^^,^^10^^,^^18-21^ thereby masking the role of non-fatal CVD diagnoses as competing events. Second, the cumulative exposure to abruption across successive pregnancies and their impact on incident maternal CVD remain less well understood. Third, a population-based study examining the impact of abruption on CVD events over an extended follow-up period in the United States is lacking.

The prevalence rate of abruption in the United States and Canada is higher than that in Denmark, Finland, Norway, Spain, and Sweden.^22^ Moreover, abruption rates have been increasing in the United States compared to those in Scandinavian populations.^22^ Furthermore, CVD mortality rates among US individuals are higher compared to similar data from Denmark, Norway, Sweden, and the United Kingdom.^23^ The difference is mainly attributed to the slowing of reductions in heart disease^24^ and a decline in stroke mortality.^25^ It is believed that predisposing behavioral risk factors for CVD (obesity, smoking, and alcohol and drug abuse) may largely account for these differences.^22^

Given the shared etiologies and overlapping risk factors between abruption and CVD, we hypothesize that individuals with pregnancies affected by abruption would experience an increased burden of all-cause mortality, non-fatal CVD events, and mortality after CVD hospitalization.

## Methods

We designed this study using the *Placental Abruption and Cardiovascular Event Risk (PACER)* cohort, which encompasses all pregnancies, deliveries (at 20 weeks or greater in gestation), hospitalizations, and mortality in New Jersey, USA.^26^ All live births and fetal deaths that occurred between 1993 and 2020 and were probabilistically linked to the delivery hospitalization, subsequent hospitalizations, and mortality files. These linkages were accomplished using the individual’s first/middle/last name, municipality, race/ethnicity, and birth date, blocking on the year of delivery. The linkage resulted in approximately 1.8 million persons with 3.1 million deliveries across all hospitals and birthing centers in New Jersey. Further details regarding the linkage algorithm and cohort description have been previously described.^26^ The *PACER* project received ethics approval and a waiver of consent from the Institutional Review Boards of Rutgers Robert Wood Johnson Medical School, New Jersey.

### Placental abruption

A clinical diagnosis of abruption includes individuals who present with bleeding accompanied by at least one of the following conditions: fetal distress, uterine tenderness, or uterine hypertonicity. If the placenta showed evidence of a tightly adherent clot consistent with retroplacental bleeding, infarctions, or other sonographic signs of abruption were present, the diagnosis of abruption was recorded.^5^^,^^27^ An abruption was determined from vital records or corresponding delivery hospitalization diagnosis codes based on the *International Classification of Diseases* (ICD), version 9 (between 1993 and 2015) and version 10 (between 2016 and 2020), described in Table S1.

### Cardiovascular disease

The primary endpoints, which included all-cause mortality and non-fatal CVD hospitalization, were examined within a transition-state modeling framework. Since the number of CVD mortality events was small, we were unable to examine this specific outcome in the transition models. Therefore, we examined all-cause mortality for the transition-state models. In secondary analyses using traditional Cox models, we examined the risks of CVD mortality, as well as mortality from heart disease and stroke. Heart disease includes ischemic heart disease, atherosclerotic heart disease, acute myocardial infarction, hypertensive heart disease, heart failure, cardiomyopathy, and cardiac arrhythmia. We examined ischemic and hemorrhagic strokes as subclassifications of stroke (Table S1). CVD mortality consisted of CVD as the underlying cause of death identified by ICD-9 and 10 codes on mortality records. Non-fatal CVD hospitalization was defined by the presence of an ICD-9 or 10 CVD diagnosis code at the time of hospitalization, regardless of whether it was the primary cause of hospitalization.

### Exclusion criteria

To prevent reverse causal bias, we excluded persons with a history of CVD hospitalization before or at the time of the first delivery, deliveries with missing abruption status (missing on birth certificate or fetal death and unlinked delivery hospitalization), and those with a history of twins and higher-order multiples in their reproductive history. The study was restricted to two non-overlapping cohorts of individuals with their first delivery only (parity 1 cohort) and those with the first two consecutive deliveries (parity 1-2 cohort). We further excluded women with a history of CVD hospitalization between the first two deliveries in the parity (1-2 cohort) to prevent reverse causal bias.

### Statistical analysis

We fitted competing-risk Cox proportional hazards regression models based on the semi-Markov multistate transition methodology to estimate the associations between abruption and mortality, as well as non-fatal CVD events. Multistate models enable the estimation of associations between exposure and the outcome of interest (eg, mortality) by partitioning risks through various pathways or transitions^28^: the effect of abruption on mortality with no intervening CVD versus the effect preceded by a non-fatal CVD hospitalization. We transformed the data into a counting process style using the *mstate* package^29^ in R (R Core Team, Vienna, Austria).

**Figure 1:**
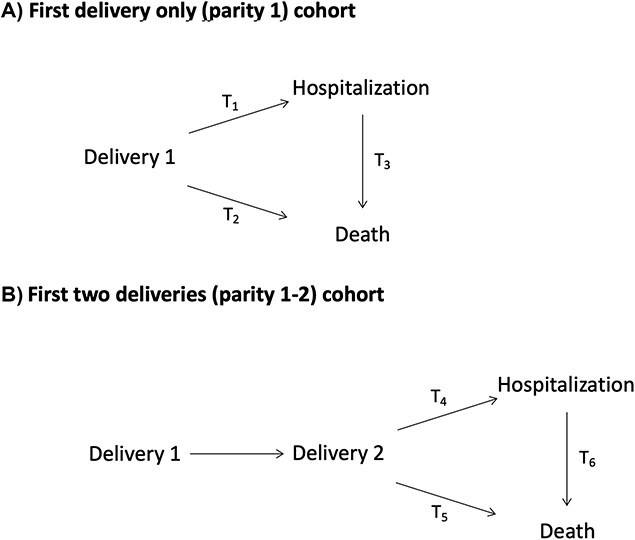
Multistate modeling framework depicting the six transition states relating placental abruption to the risk of cardiovascular disease events (mortality and non-fatal hospitalizations). The transitions are depicted for the parity 1 cohort and parity 1-2 cohorts: *Placental Abruption and Cardiovascular Event Risk* (*PACER*), 1993 to 2020.

We examined the effect of abruption on the risk of incident CVD hospitalization and mortality in two non-overlapping cohorts: those who had only delivered once during the study period (parity 1) and those with two or more deliveries (parity 1-2). We modeled six transitions: (1) first delivery to first CVD hospitalization, (2) first delivery to mortality, (3) first delivery to mortality after first CVD hospitalization, (4) second delivery to first CVD hospitalization, (5) second delivery to mortality, and (6) second delivery to mortality with a preceding CVD hospitalization. The first three transitions pertain only to the parity 1 cohort, while the latter three transitions pertain to the parity 1-2 cohort (Figure 1). In all models, the start time was defined as the date of the first delivery, and the follow-up time was determined between the delivery dates and the occurrence of incident CVD hospitalization, death, CVD hospitalization to death, or until the end of the study period (December 31, 2020), if no event occurred.

The linkage proportions for live births and hospitalizations, and fetal deaths and hospitalizations were 92.4% and 70.7%, respectively. To account for unlinked records that may lead to selection bias and affect the association between abruption and CVD, we derived inverse probability of selection weights^26^ and applied them to all analyses.

### Covariates

We estimated the unadjusted and confounder-adjusted HRs with 95% CIs. The following confounders were adjusted for maternal age, mother’s education (<9, 9-12, 13-16, and 17 or more years of completed schooling), insurance coverage (Medicare, Medicaid, private, self-pay, and others), and marital status (single or married) as a proxy for the social determinants of health. We also adjusted for tobacco use before or during pregnancy (yes or no), hypertensive disorders of pregnancy (chronic hypertension, gestational hypertension, preeclampsia, eclampsia), pregestational diabetes, gestational diabetes, and year of delivery. We additionally adjusted for the mother's race/ethnicity (non-Hispanic Black, Hispanic, non-Hispanic White, and other races [Indian (North American, Central American, South American, Eskimo, and Aleut), Chinese, Japanese, Hawaiian, Filipino, other Asians (Pakistani, Bangladeshi, Cambodian, Thai), unknown Asian Indian, Korean, Samoan, Vietnamese, Guamian]) as a marker for exposure to racism. These confounders were specific to each delivery, depending on the transitions. For abruption as a risk factor in the second pregnancy, we adjusted for the interpregnancy interval (IPI), defined as the latency between the first delivery and the date of delivery of the second pregnancy minus the gestational age. Maternal age, IPI, and delivery year were modeled as continuous variables; quadratic terms for age and IPI were included in the models, centered after subtracting their respective mean values.

### Unmeasured confounding bias

We assessed how unmeasured confounding may have influenced the associations between abruption and CVD hospitalizations and mortality in the multistate models. For an unmeasured confounder to fully explain away the confounder-adjusted HRs, the exposure-unmeasured confounder (HR_EU_) and unmeasured confounder-outcome (HR_UD_) would both have to be at least as strong as the observed confounder-adjusted HR.^30^ Under this extreme scenario, the joint bounding bias factor was derived as (HR_EU_ × HR_UD_)/(HR_EU_ + HR_UD_ − 1). We then divided the observed HR (and its corresponding 95% CI) by the bias factor to derive the true causal HR adjusted for both the observed and unmeasured confounders.^31^ The advantage of this method is that it does not impose any assumptions regarding HR_EU_ and HR_UD_.

### Missing data

Since data were missing in covariates (Table S2), we imputed missing data using the fully conditional specification approach (25 imputed data sets were created). We fit models for the 25 imputed data sets and combined the results using Rubin’s rule.^32^

### Sensitivity analysis

We fit traditional Cox proportional hazards models (ignoring the transition states) to examine all-cause mortality, CVD mortality, and incident non-fatal CVD hospitalizations as independent outcomes. These models were adjusted for the same confounders listed above by incorporating missing data through the multiple imputation methodology and weighting for missing record linkages.

### Exposure misclassification bias assessment

We performed a quantitative bias analysis to address potential misclassification of exposure.^33^ This was based on non-differential exposure misclassification by assuming that the sensitivity and specificity of abruption were 0.65 and 0.99, respectively.^11^^,^^27^ Under a uniform distribution and using record-level data, we estimated the bias-corrected median HR (95% CI) based on the traditional Cox model for the associations between abruption and non-fatal CVD hospitalizations, as well as all-cause mortality, across 1000 replications.^34^

## Results

### Characteristics of the total cohort

There were 1 794 364 persons with 2 874 671 singleton deliveries, resulting in an abruption proportion of 1% (*n* = 28 913) across the entire study period from 1993 to 2020. Abruption rates gradually increased with parity, and rates were higher among people who used tobacco, those with federal insurance coverage (Medicare-Medicaid) plans, and those with hypertensive disorders of pregnancy (Table S2).

In 27.3 million person-years of follow-up (median follow-up of 16.0 years, range 0-28), the all-cause mortality rates among the abruption and non-abruption groups were 106 and 61 per 100 000 person-years, respectively (Table 1). Similarly, the CVD, heart disease, and stroke mortality rates, as well as rates of incident non-fatal CVD hospitalizations, were also higher in the abruption than the non-abruption groups.

**Table 1:** Rates of cardiovascular mortality and incident non-fatal cardiovascular events over the 28-year follow-up period with and without placental abruption for all singleton deliveries: *Placental Abruption and Cardiovascular Event Risk* (*PACER*), 1993 to 2020.

	**No placental abruption (*n* = 1 766 075)**			**Placental abruption (*n* = 28 289)**		
	**Total person-years**	**Median (IQR) person-years**	**Number of events (incidence rate per 100 000 person-years)**	**Total person-years**	**Median (IQR) person-years**	**Number of events (incidence rate per 100 000 person-years)**
**Mortality**						
All causes	27 304 661	16.0 (8.5-22.9)	16 622 (61)	460 531	17.1 (9.9-23.4)	489 (106)
Cardiovascular disease	27 090 698	16.0 (8.5-22.9)	1647 (6)	454 199	17.1 (9.9-23.4)	53 (12)
Heart disease	27 083 999	16.0 (8.5-22.9)	1211 (4)	453 885	17.1 (9.9-23.4)	33 (7)
Stroke	27 070 453	16.0 (8.5-22.9)	436 (2)	453 650	17.1 (9.9-23.4)	20 (4)
**Non-fatal incident cardiovascular hospitalizations**						
Cardiovascular disease	26 423 146	15.5 (8.1-22.4)	81 472 (308)	440 189	16.4 (9.4-22.9)	1795 (408)
Heart disease	26 295 534	15.5 (8.1-22.4)	70 738 (269)	437 183	16.4 (9.4-22.9)	1542 (353)
Stroke	25 653 516	15.6 (8.2-22.6)	12 042 (47)	423 410	16.6 (9.5-23.1)	297 (70)

Abbreviations: CVD, cardiovascular disease; IQR, interquartile range.

Placental abruption status is only considered for all singleton deliveries until the first hospitalization for cardiovascular disease. 1 794 364 persons contributed to 2 874 671 singleton deliveries without CVD before or at the time of first delivery in the PACER study period.

### CVD risks in the first and first two deliveries

We examined rates of incident CVD events and all-cause and CVD-related mortality with abruption among persons in their first delivery (Table 2). All-cause mortality rates among the abruption and non-abruption groups were 103 and 66 per 100 000 person-years, and the corresponding rates of CVD-related mortality were 16 and 6 per 100 000 person-years, respectively. Among those with two deliveries (parity 1-2), CVD mortality rates, as well as incident non-fatal CVD hospitalizations, progressively increased with the number of abruptions.

**Table 2:** Rates of cardiovascular mortality and incident non-fatal cardiovascular events over the 28-year follow-up period based on placental abruption in the first delivery, and in the first and second singleton deliveries: *Placental Abruption and Cardiovascular Event Risk* (*PACER*), 1993 to 2020.

	**First delivery (Parity 1)**		**First two deliveries (Parity 1 and 2)**			
	**No abruption**	**Abruption**	**No abruption at either delivery**	**Abruption at first delivery only**	**Abruption at second delivery only**	**Abruption at both deliveries**
Number of subjects	573 582	6151	588 412	4714	5203	167
Total person-years	7 404 614	79 735	9 502 880	75 912	83 415	2532
Number of events (Incidence rate per 100 000 person-years)						
Mortality						
All-cause	4902 (66)	82 (103)	4038 (42)	46 (61)	68 (82)	–^b^
Cardiovascular disease	443 (6)	13 (16)	331 (3)	–^b^	6 (7)	–^b^
Incident non-fatal cardiovascular hospitalizations						
Latency: Delivery to non-fatal CVD hospitalization^**a**^	10.3 (4.8-16.2)	10.8 (5.1-16.8)	10.9 (5.5-16.4)	10.8 (4.8-15.5)	10.3 (5.4-15.8)	8.8 (5.2-12.0)
Cardiovascular disease (any)	19 244 (260)	268 (336)	29 628 (312)	281 (370)	363 (435)	13 (513)
Heart disease	16 868 (228)	235 (295)	26 132 (275)	248 (327)	322 (386)	12 (474)
Stroke	2697 (36)	41 (51)	3847 (40)	42 (55)	46 (55)	–^b^
Non-fatal CVD hospitalizations to all-cause mortality						
Person-years	7 382 008	79 372	9 480 815	75 605	83 036	2506
Latency: Non-fatal CVD to all-cause mortality^**a**^	0.5 (0.0-4.3)	0.8 (0.0-10.3)	0.6 (0.0-3.9)	1.0 (0.1-3.2)	4.2 (0.0-8.3)	2.1 (0.4-3.7)
Cardiovascular disease to all-cause mortality	2016 (27)	31 (39)	1689 (18)	25 (33)	31 (37)	–^b^
Heart disease to all-cause mortality	1787 (24)	26 (33)	1457 (15)	22 (29)	28 (34)	–^b^
Stroke to all-cause mortality	291 (4)	7 (9)	287 (3)	6 (8)	–^b^	–^b^

Abbreviation: CVD, cardiovascular disease.

^a^Data are reported as median (interquartile range) in years.

^b^Data are suppressed when the number of events is <5.

We examined the unadjusted (Table S3) and confounder-adjusted (Table 3) association between abruption and the risk of CVD events based on non-fatal heart disease and stroke hospitalization and mortality transitions. The corresponding Kaplan–Meier cumulative survival function for all transition states over the follow-up period, based on abruption status, is shown in Figures S1 and S2. Survival probabilities were lower among abruptions compared to non-abruption deliveries. The likelihood of survival was lowest for non-fatal CVD hospitalization to death for individuals with abruption in the first delivery (parity 1) or first two deliveries (parity 1-2).

**Table 3:** Associations between placental abruption (in the first delivery and the first two deliveries) and risks of cardiovascular events (mortality and incident non-fatal complications) over the 28-year follow-up period: *Placental Abruption and Cardiovascular Event Risk (PACER)*, 1993 to 2020.

	**Abruption in the first delivery (Parity 1)** ^**a**^	**Abruption in the first two deliveries (Parity 1 and 2)** ^**b**^		
		**Abruption in the first delivery only**	**Abruption in the second delivery only**	**Abruption in both deliveries**
Cardiovascular disease events				
Delivery to non-fatal cardiovascular disease	1.27 (1.11-1.46)	1.04 (0.90-1.19)	1.17 (1.03-1.33)	1.11 (0.60-2.06)
Delivery to all-cause death	1.77 (1.28-2.44)	0.95 (0.62-1.47)	1.43 (1.00-2.04)	3.19 (0.74-13.78)
Non-fatal cardiovascular disease to all-cause death	1.52 (0.98-2.37)	1.52 (0.98-2.37)	1.16 (0.79-1.70)	2.52 (0.57-11.19)
Heart disease events				
Delivery to non-fatal heart disease	1.27 (1.10-1.48)	1.05 (0.91-1.21)	1.15 (1.01-1.32)	1.17 (0.62-2.23)
Delivery to all-cause death	1.73 (1.25-2.39)	0.97 (0.65, 1.45)	1.38 (0.98-1.95)	2.79 (0.64-12.10)
Non-fatal heart disease to all-cause death	1.55 (0.96-2.51)	1.55 (0.96, 2.51)	1.18 (0.79-1.77)	2.95 (0.67-12.95)
Stroke events				
Delivery to non-fatal stroke	1.37 (1.00-1.88)	1.07 (0.76, 1.50)	1.28 (0.91-1.80)	
Delivery to all-cause death	1.62 (1.22-2.15)	1.21 (0.89, 1.65)	1.46 (1.13-1.90)	
Non-fatal stroke to all-cause mortality	1.65 (0.87-3.11)	1.65 (0.87, 3.11)	1.19 (0.44-3.25)	

Table entries denote the adjusted HR and 95% CI based on the multi-state Cox proportional hazards model. The Cox proportional hazards model was weighted by inverse probability weights to account for missing linkage at first delivery. All HRs were estimated from results pooled from models based on 25 multiply imputed datasets, with no abruption in the first delivery (parity 1 analysis) or no abruption in the first and second delivery (parity 1 and 2 analysis) as the reference.

^a^HRs were adjusted for confounding effects of the mother’s age, mother’s race (non-Hispanic Black, Hispanic, non-Hispanic White, and other races [Indian {North American, Central American, South American, Eskimo, and Aleut}, Chinese, Japanese, Hawaiian, Filipino, other Asian [Pakistani, Bangladeshi, Cambodian, Thai], unknown, Asian Indian, Korean, Samoan, Vietnamese, Guamian), marital status, mother’s education, insurance, smoking status, hypertensive disorders of pregnancy (chronic hypertension, gestational hypertension, preeclampsia, and eclampsia), pregestational diabetes, and year of delivery of the first pregnancy.

^b^HRs were adjusted for confounding effects of the mother’s age, mother’s race (non-Hispanic Black, Hispanic, non-Hispanic White, and other races [Indian {North American, Central American, South American, Eskimo, and Aleut}, Chinese, Japanese, Hawaiian, Filipino, other Asian [Pakistani, Bangladeshi, Cambodian, Thai], Unknown, Asian Indian, Korean, Samoan, Vietnamese, Guamian), marital status, mother’s education, insurance, smoking status, hypertensive disorders of pregnancy (chronic hypertension, gestational hypertension, preeclampsia, and eclampsia), pregestational diabetes, interpregnancy interval, and year of delivery of the first and second pregnancies.

**Figure 2:**
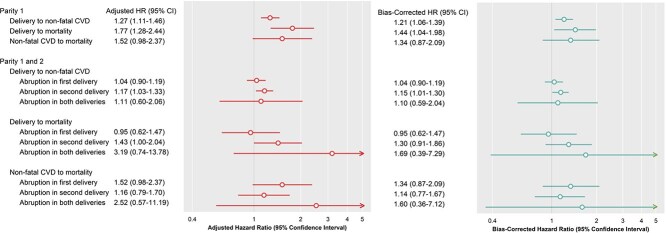
Forest plot depicting the associations (adjusted HR and 95% CI) between placental abruption and all-cause mortality and non-fatal cardiovascular hospitalization: *Placental Abruption and Cardiovascular Event Risk* (*PACER*), 1993 to 2020. *Legend*: Associations are shown as adjusted HRs (95% CIs) in the left panel, and the bias-corrected HRs (95% CIs) in the right panel. All associations are shown for the first delivery and the first two deliveries.

In a multistate model, among the first deliveries complicated by abruption, the risks of delivery to non-fatal CVD hospitalization, delivery to all-cause mortality, and all-cause mortality after hospitalization for non-fatal CVD events were 1.27-, 1.77-, and 1.52-fold higher in those who gave birth after abruption than in individuals who did not experience abruption (Figure 2). Among the first two deliveries, abruption in both deliveries was associated with higher risks for all three transition states. Compared to no abruptions in the first two deliveries (parity 1 and 2), the adjusted HRs for delivery to non-fatal CVD and from delivery to all-cause mortality increased for abruption in the first delivery (parity 1), abruption in the second delivery (parity 2), and abruption in both deliveries. However, the corresponding risks for non-fatal CVD to all-cause death were modestly lower among abruptions in the second delivery.

We derived estimates of the effect of abruption on all-cause mortality and non-fatal CVD hospitalization, as described in Table 3, following corrections for unmeasured confounding bias (Figure 2). Although the strength of the HRs denoting the association between abruption and risks of all-cause mortality, as well as non-fatal CVD hospitalization, were slightly attenuated compared to the corresponding confounder-adjusted estimates, these bias corrections still indicate increased risks of outcomes.

### Sensitivity analysis

Compared to persons without abruption, the rates of mortality and non-fatal CVD hospitalization among those with abruption were higher (Table S4). In the traditional Cox modeling approach, the risks before and after accounting for confounders showed that all-cause mortality and non-fatal heart disease and stroke hospitalizations progressively increased with recurrent abruptions (Tables S5 and S6).

We applied the corrections for exposure misclassification bias to the estimates based on the confounder-adjusted traditional Cox models for abruption at first delivery (Table S6). The bias-corrected hazard ratios (HRs) for all-cause mortality and non-fatal CVD hospitalizations were 2.18 (95% CI, 1.05-4.80) and 1.64 (95% CI, 0.83-3.49), respectively.

## Discussion

**Figure 3:**
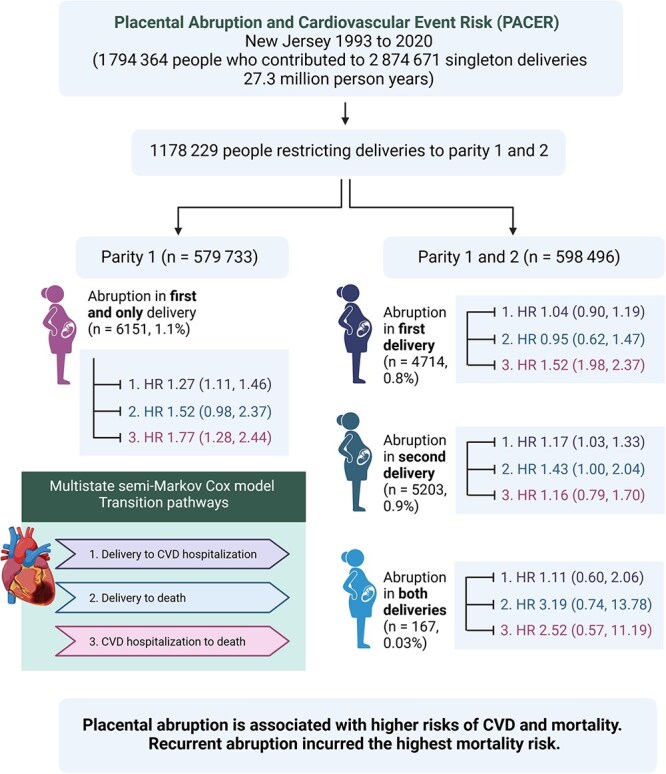
Graphical abstract that provides a visual summary of the study: *Placental Abruption and Cardiovascular Event Risk* (*PACER*), 1993 to 2020.

This population-based study of nearly 1.8 million individuals, including 2.9 million singleton deliveries, with a median follow-up of 16 years, provides new insights into how and to what extent CVD and mortality risks are associated with placental abruption. This study documents the increased burden of all-cause mortality, non-fatal incident CVD events, and mortality after an intervening non-fatal CVD hospitalization among individuals with pregnancies complicated by time-varying abruption across pregnancies. CVD risks were higher, with recurrent abruptions carrying the highest risks of all-cause mortality and non-fatal CVD hospitalization (Figure 3). These strong associations, even following adjustments for hypertensive disorders, tobacco use, and other confounders, as well as correcting for unmeasured confounding and exposure misclassification biases, suggest that abruption may be an independent risk factor for CVD risk.

### Biological pathways

At least three pathways may help explain these associations. First, both abruption and CVD risks share similar epidemiologic risk factors and clinical profiles,^17^ including advanced age, tobacco use, overweight or obesity, dyslipidemia, renal dysfunction, and metabolic disorders.^35^ Second, inadequate spiral artery remodeling, endothelial cell dysfunction, poor placental perfusion, and oxidative stress are the chief causes of placental dysfunction^36^ and uteroplacental ischemia.^16^ These subclinical markers may increase the burden of vascular stress and inflammation, leading to impaired vascular perfusion, which is strongly implicated in CVD risk.^37^ These processes result in defective physiological changes in the myometrial segment of uteroplacental spiral arteries, which, in turn, lead to atherosis in their proximal segment causing obstructive vascular pathology.^38^ The commonality in these pathophysiologic processes between abruption and CVD suggests a shared etiology.^19^ Third, pregnancy itself increases vascular stress and may accelerate the development of cardiometabolic risk factors after delivery and enhance CVD risk.^39^^,^^40^

### Comparison with other studies

Only a handful of studies have investigated the relationship between placental abruption and CVD outcomes using traditional Cox models. A meta-analysis^11^ reported that abruption was associated with higher mortality from heart disease (risk ratio [RR], 2.64; 95% CI, 1.57-4.44; 5 studies) and stroke (RR, 1.70; 95% CI, 1.19-2.42; 3 studies). The risk of non-fatal CVD associated with abruption was 1.32 (95% CI, 0.91-1.92; 5 studies). DeRoo and colleagues^7^ examined CVD mortality rates in the medical birth registry and death registries of over two million persons with first and first two singleton births between 1967 and 2002 in Norway and 1973 and 2003 in Sweden (mean follow-up, 23 years; range, 1-42 years). They showed that those who experienced abruption in their first pregnancy had a higher risk of all-cause mortality (HR, 1.2; 95% CI, 1.0-1.3) and CVD mortality risk (HR 1.8; 95 % CI, 1.3-2.4). They reported increased CVD mortality risk for persons with one lifetime pregnancy compared to those who had multiple pregnancies and abruption only in the first pregnancy, and higher risks of all-cause and CVD mortality with recurrent abruptions. Similar associations were reported in two population-based cohort studies in Denmark.^18^^,^^19^ Our study demonstrated similar trends, but we were unable to describe the association between recurrent abruptions and mortality and CVD morbidity risks. Multistate modeling uncovers the heterogeneity in all-cause mortality risk with and without a preceding CVD hospitalization associated with abruption.

### Strengths and limitations

A fundamental drawback of virtually all previous explorations of obstetrical complications as risk factors for CVD is the likely underestimation of risks. This study is one of the first to document the underlying heterogeneity in these risks, utilizing time-varying exposures and covariates between pregnancies within a multistate modeling framework to elucidate the relationship between abruption and CVD morbidity and mortality. Other strengths include the application of the inverse probability weighting method to account for unlinked delivery hospitalizations, adjustment for numerous confounders, imputation of missing covariate information, and correction for unmeasured confounding. An evaluation of exposure misclassification of abruption suggests that the misclassification strengthened the association for both all-cause mortality and non-fatal CVD hospitalizations.

The interpretation of the findings should take into account several limitations. The cohort's maximum follow-up is 28 years after delivery, and individuals may not yet have experienced CVD events. We were unable to adjust for pre pregnancy body-mass index and drug and alcohol use since these data are only available in recent years. However, bias corrections for such unmeasured confounders show that the associations between abruption and increased CVD risk persist. Some of the covariate distributions differed between the linked and unlinked deliveries with hospitalization records.^26^ For example, the proportions of non-Hispanic Blacks, Hispanics, low education levels, and unmarried persons were higher among the unlinked than the linked records. We address this potential bias by incorporating the inverse probability weighting strategy for all analyses. The *PACER* lacked data on objective clinical markers (such as blood pressure and lipid profiles). These clinical markers will be helpful for improved risk characterization and prediction of abruption, and future studies may consider evaluating these markers.

Abruption is strongly implicated in substantial risks of disseminated intravascular coagulation^41^ and postpartum hemorrhage.^42^ The traumatic experience of an unfavorable pregnancy or the abruption itself, warranting a hysterectomy,^5^ may prevent individuals from going on to have a second pregnancy. Thus, the cohort of persons with only their first birth may be unique and likely biased by selection (selective fertility^43^). For the cohort of individuals with their first two deliveries (parity 1-2), the associations are generalizable to those who survive and have two births.

## Conclusions

As one of the largest cohorts in the US, this study underscores the substantial burden that placental abruption places on the cardiovascular system along the life course. There is increasing awareness of the enhanced CVD risks among those whose pregnancies are complicated by chronic hypertension,^44^ preeclampsia,^45^^,^^46^ gestational diabetes,^47^ and preterm delivery.^48-50^ The consequence of abruption is that it is associated with maternal and fetal risks that are far stronger than any other obstetrical complications. Despite the decline in stroke and heart disease mortality,^24^^,^^25^^,^^51^ it is plausible that abruption may unmask the burden of CVD events. Reducing unhealthy behaviors through lifestyle modifications, increasing physical activity, modifying known CVD risk factors such as hypertension and hyperlipidemia, and postpartum CVD follow-up may be of even greater value following a pregnancy complicated by abruption. The accumulating evidence underscores the inclusion of abruption as yet another obstetrical complication with associated cardiovascular risk.

## Supplementary Material

Web_Material_kwaf289

## Data Availability

The *PACER* project was developed after signed data use agreements between the Department of Health, State of New Jersey, and Rutgers Biomedical and Health Sciences, NJ. Due to data privacy issues, we are unable to make this data publicly available.
