# Supplementary material for: Cardiovascular disease and mortality following placental abruption
Source: Am J Epidemiol. 2026 Jan 20;195(6):1546–54. doi: 10.1093/aje/kwaf289 (PMC13231852; doi:10.1093/aje/kwaf289)
Supplement: Web_Material_kwaf289 [file web_material_kwaf289.docx]

**Cardiovascular Disease and Mortality following Placental Abruption**

Cande V. Ananth, Rachel Lee, Linda Valeri,

Sonia M. Grandi, Todd Rosen, William J. Kostis,
for the *PACER* Study Investigators

**Supplemental Figures and Tables**

|  | **Title** |
| --- | --- |
|  |  |
| **Figure S1** | Kaplan-Meier curve showing the cumulative survival and hospitalization among first deliveries (parity 1) with and without a diagnosis of placental abruption: *Placental Abruption and Cardiovascular Event Risk (PACER)*, 1993 to 2020  *Legend*: Transition 1 refers to delivery to CVD hospitalization. Transition 2 relates to delivery to all-cause mortality. Transition 3 refers to CVD hospitalization to all-cause mortality after delivery. Solid lines refer to abruption, and dotted lines refer to non-abruption. The panel on the right represents transitions 1 and 2 with the scale on the y-axis expanded for easier interpretation |
|  |  |
| **Figure S2** | Kaplan-Meier curve showing the cumulative survival and hospitalization among the first two deliveries (parity 1 and 2) with and without a diagnosis of placental abruption: *Placental Abruption and Cardiovascular Event Risk (PACER)*, 1993 to 2020  *Legend*: Transition 1 refers to delivery to CVD hospitalization. Transition 2 refers to delivery to all-cause mortality. Transition 3 refers to CVD hospitalization to all-cause mortality after delivery. Solid lines refer to abruption, and dotted lines refer to non-abruption. The panel on the right represents transitions 1 and 2 with the scale on the y-axis expanded for easier interpretation |
|  |  |
|  |  |
| **Table S1** | International Classification of Diseases (ICD) 9 and 10 codes for  *Placental Abruption and Cardiovascular Event Risk (PACER)*, 1993 to 2020 |
|  |  |
| **Table S2** | Distribution of maternal sociodemographic characteristics in relation to placental abruption in singletons: *Placental Abruption and Cardiovascular Event Risk (PACER)*, 1993-2020 |
|  |  |
| **Table S3** | Unadjusted associations between placental abruption and cardiovascular events (mortality and incident non-fatal complications) among the first singleton delivery only and time-varying placental abruption in the first and second singleton deliveries using multi-state models: *Placental Abruption and Cardiovascular Event Risk (PACER)*, 1993-2020 |
|  |  |
| **Table S4** | Rates for placental abruption and cardiovascular events (mortality and incident non-fatal complications) among the first singleton delivery only and time-varying placental abruption in the first and second singleton deliveries: *Placental Abruption and Cardiovascular Event Risk (PACER)*, 1993-2020 |
|  |  |
| **Table S5** | Unadjusted associations between placental abruption and cardiovascular events (mortality and incident non-fatal complications) among the first singleton delivery only and time-varying placental abruption in the first and second singleton deliveries based on traditional Cox proportional hazards regression models: *Placental Abruption and Cardiovascular Event Risk (PACER)*, 1993-2020 |
|  |  |
| **Table S6** | Adjusted associations between placental abruption and cardiovascular events (mortality and incident non-fatal complications) among the first singleton delivery only and time-varying placental abruption in the first and second singleton deliveries based on traditional Cox proportional hazards regression models: *Placental Abruption and Cardiovascular Event Risk (PACER)*, 1993-2020 |

**Figure S1**

**Kaplan-Meier curve showing the cumulative survival and hospitalization among first deliveries (parity 1) with and without a diagnosis of placental abruption: *Placental Abruption and Cardiovascular Event Risk (PACER)*, 1993 to 2020**

*Legend*: Transition 1 refers to delivery to CVD hospitalization. Transition 2 relates to delivery to all-cause mortality. Transition 3 refers to CVD hospitalization to all-cause mortality after delivery. Solid lines refer to abruption, and dotted lines refer to non-abruption. The panel on the right represents transitions 1 and 2 with the scale on the y-axis expanded for easier interpretation.

**
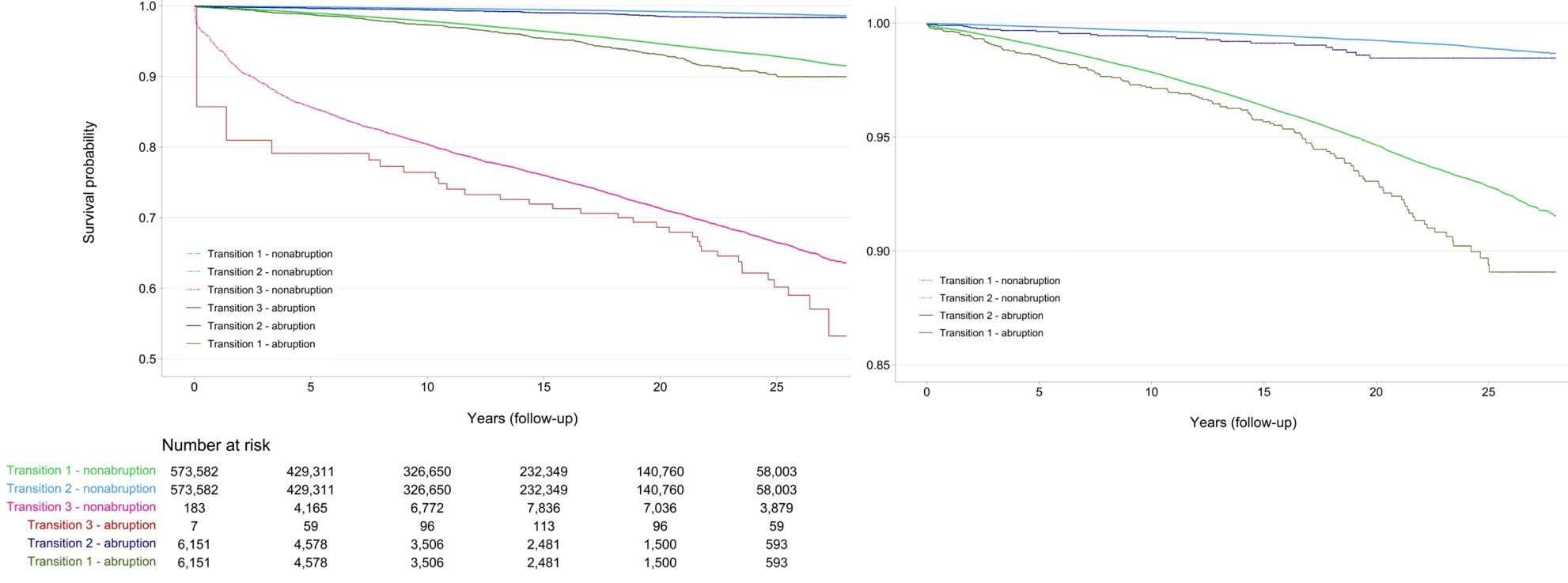
**

**Figure S2**

**Kaplan-Meier curve showing the cumulative survival and hospitalization among the first two deliveries (parity 1 and 2) with and without a diagnosis of placental abruption: *Placental Abruption and Cardiovascular Event Risk (PACER)*, 1993 to 2020**

*Legend*: Transition 1 refers to delivery to CVD hospitalization. Transition 2 refers to delivery to all-cause mortality. Transition 3 refers to CVD hospitalization to all-cause mortality after delivery. Solid lines refer to abruption, and dotted lines refer to non-abruption. The panel on the right represents transitions 1 and 2 with the scale on the y-axis expanded for easier interpretation.

**
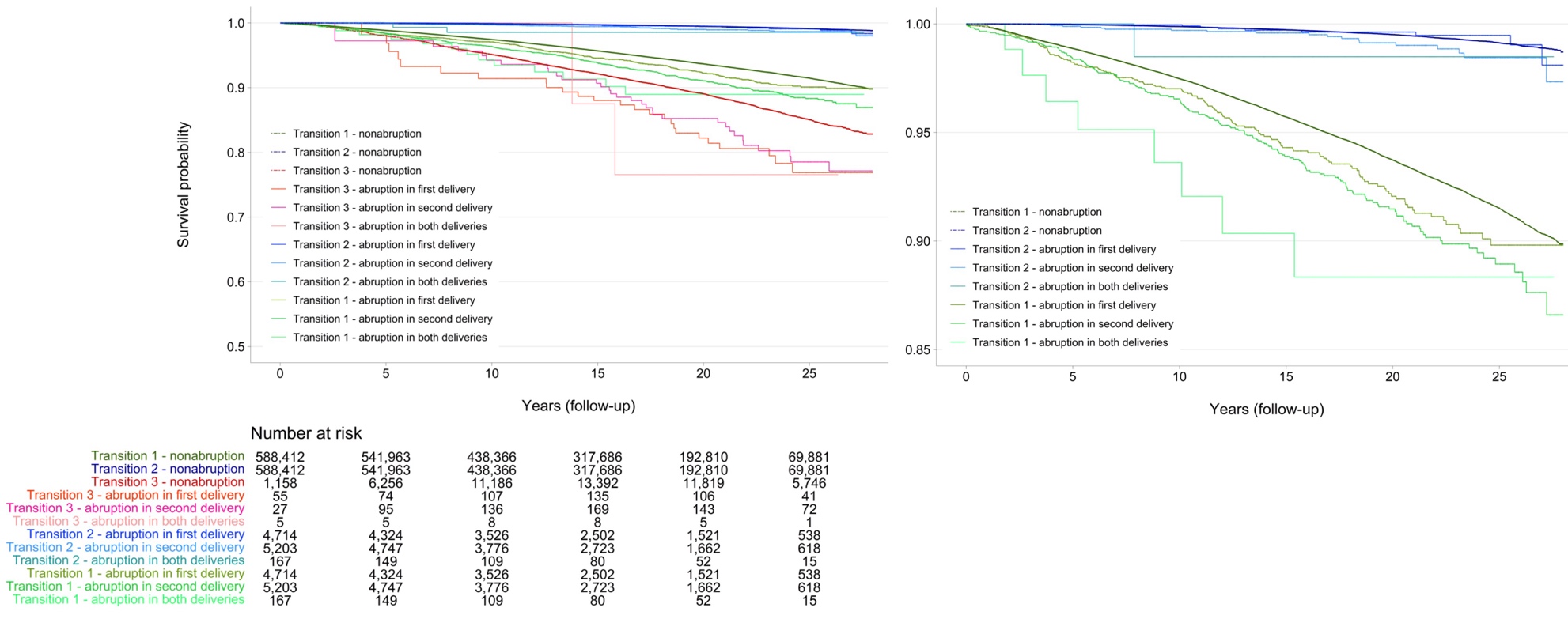
**

**Table S1**

***International Classification of Diseases* (ICD) 9 and 10 codes for
*Placental Abruption and Cardiovascular Event Risk (PACER)*, 1993 to 2020**

|  | **ICD-9 Hospitalization (1993 to 2015)**  **Mortality (1993 to 1999)** | **ICD-10 Hospitalization (2016 to 2020)**  **Mortality (2000 to 2020)** |
| --- | --- | --- |
|  |  |  |
| **Exposure** |  |  |
| Placental abruption | 641.2 | O45 |
|  |  |  |
| **Index Event** |  |  |
| Cardiovascular disease (any) | 402, 410-414, 425, 427, 428, 430-438, 440-449 | I11, I20-I25, I42, I46-I50, I60-I70 |
| Heart disease (any) | 398.91, 402, 410-414, 425, 427, 428, 440-449 | I09.81, I11, I20-I25, I42, I46-I50, I70 |
| Ischemic heart disease | 410-414 | I20-I25 |
| Atherosclerotic heart disease | 440-449 | I70 |
| Acute myocardial infarction | 410 | I21, I22 |
| Hypertensive heart disease | 402 | I11 |
| Heart failure | 428 | I50 |
| Cardiomyopathy | 425 | I42 |
| Cardiac arrhythmias | 427 | I46-I49 |
|  |  |  |
| Stroke (any) | 430-438 | I60-I69 |
| Ischemic stroke | 430-432 | I60-I62, I69 |
| Hemorrhagic stroke | 433-437 | I63, I65-I67 |
|  |  |  |
| **Maternal risk factors** |  |  |
| Maternal smoking | 649.0 | O99.33 |
|  |  |  |
| **Obstetrical and labor and delivery complications** | |  |
| Pre-pregnancy diabetes | 250, 648.0 | E08-E13, O24.0, O24.1, O24.3, O24.8 |
| Gestational diabetes | 648.8 | O24.4, O24.9 |
| Chronic hypertension | 642.00-642.04, 642.20-642.24 | O10.0, O10.4, O10.9 |
| Gestational hypertension | 642.30-642.34 | O13 |
| Preeclampsia without severe features | 642.40-642.44 | O14.0, O14.9 |
| Preeclampsia with severe features | 642.50-642.54 | O14.1, O14.2 |
| Eclampsia | 642.60-642.64 | O15 |
| Superimposed preeclampsia | 642.70-642.74 | O11 |
| Unspecified hypertension | 642.90-642.94 | O16 |
|  |  |  |

**Table S2**

**Distribution of maternal sociodemographic characteristics in relation to placental abruption in singletons:**

***Placental Abruption and Cardiovascular Event Risk (PACER)*, 1993-2020**

|  | **All deliveries** | | | **First delivery (Parity 1)** | | | **Second delivery (Parity 2)** | | |
| --- | --- | --- | --- | --- | --- | --- | --- | --- | --- |
|  | **Total cohort**  **No. (%_col_)** | **Abruption**  **No. (%_col_)** | **Abruption**  **(%_row_)** | **Total cohort**  **No. (%_col_)** | **Abruption**  **No. (%_col_)** | **Abruption**  **(%_row_)** | **Total cohort**  **No. (%_col_)** | **Abruption**  **No. (%_col_)** | **Abruption**  **(%_row_)** |
|  |  |  |  |  |  |  |  |  |  |
| Total deliveries | 2,874,671 (100.0) | 28,913 (100.0) | (1.0) | 1,178,229 (100.0) | 11,302 (100.0) | (0.9) | 598,496 (100.0) | 5,370 (100.0) | (0.9) |
|  |  |  |  |  |  |  |  |  |  |
| Delivery year |  |  |  |  |  |  |  |  |  |
| 1993-1995 | 326,461 (11.4) | 3,155 (10.9) | (1.0) | 141,951 (12.0) | 1,286 (11.7) | (0.9) | 12,278 (2.1) | 113 (2.1) | (0.9) |
| 1996-2000 | 528,835 (18.4) | 5,539 (19.2) | (1.0) | 219,357 (18.6) | 2,049 (18.6) | (0.9) | 104,510 (17.5) | 869 (16.2) | (0.8) |
| 2001-2005 | 547,350 (19.0) | 5,344 (18.5) | (1.0) | 221,610 (18.8) | 2,036 (18.5) | (0.9) | 126,632 (21.2) | 1,105 (20.6) | (0.9) |
| 2006-2010 | 527,103 (18.3) | 5,167 (17.9) | (1.0) | 213,853 (18.2) | 2,010 (18.2) | (0.9) | 125,337 (20.9) | 1,131 (21.1) | (0.9) |
| 2011-2015 | 487,203 (16.9) | 4,727 (16.3) | (1.0) | 200,105 (17.0) | 1,824 (16.5) | (0.9) | 117,213 (19.6) | 1,028 (19.1) | (0.9) |
| 2016-2020 | 457,719 (15.9) | 4,981 (17.2) | (1.1) | 181,353 (15.4) | 1,827 (16.6) | (1.0) | 112,526 (18.8) | 1,124 (20.9) | (1.0) |
|  |  |  |  |  |  |  |  |  |  |
| Maternal age (years) |  |  |  |  |  |  |  |  |  |
| Mean (SD) | 29.8 ± 6.0 | 29.8 ± 6.0 | 30.2 ± 6.2 | 27.7 ± 6.0 | 27.7 ± 6.0 | 28.1 ± 6.4 | 30.5 ± 5.4 | 30.5 ± 5.4 | 30.3 ± 5.9 |
| <20 | 169,826 (5.9) | 1,711 (5.9) | (1.0) | 139,732 (11.9) | 1,303 (11.8) | (0.9) | 16,344 (2.7) | 221 (4.1) | (1.4) |
| 20-24 | 461,257 (16.0) | 4,608 (15.9) | (1.0) | 250,798 (21.3) | 2,271 (20.6) | (0.9) | 90,354 (15.1) | 945 (17.6) | (1.0) |
| 25-29 | 743,409 (25.9) | 6,872 (23.8) | (0.9) | 334,518 (28.4) | 2,803 (25.4) | (0.8) | 142,312 (23.8) | 1,172 (21.8) | (0.8) |
| 30-34 | 902,857 (31.4) | 8,756 (30.3) | (1.0) | 310,221 (26.3) | 2,973 (26.9) | (1.0) | 218,540 (36.5) | 1,755 (32.7) | (0.8) |
| 35-39 | 483,611 (16.8) | 5,373 (18.6) | (1.1) | 115,261 (9.8) | 1,315 (11.9) | (1.1) | 110,681 (18.5) | 1,046 (19.5) | (0.9) |
| 40-44 | 101,446 (3.5) | 1,384 (4.8) | (1.4) | 23,322 (2.0) | 309 (2.8) | (1.3) | 18,267 (3.1) | 202 (3.8) | (1.1) |
| ≥45 | 6,256 (0.2) | 109 (0.4) | (1.7) | 1,937 (0.2) | 30 (0.3) | (1.5) | 757 (0.1) | 12 (0.2) | (1.6) |
| Missing | 6,009 (0.2) | 100 (0.3) | (1.7) | 2,440 (0.2) | 28 (0.3) | (1.1) | 1,241 (0.2) | 17 (0.3) | (1.4) |
|  |  |  |  |  |  |  |  |  |  |
| Parity |  |  |  |  |  |  |  |  |  |
| 1 | 1,191,873 (41.5) | 11,177 (38.7) | (0.9) | 1,178,229 (100.0) | 11,032 (100.0) | (0.9) |  |  |  |
| 2 | 965,939 (33.6) | 9,014 (31.2) | (0.9) |  |  |  | 598,496 (100.0) | 5,370 (100.0) | (0.9) |
| ≥3 | 683,060 (23.8) | 8,604 (29.8) | (1.3) |  |  |  |  |  |  |
| Missing | 33,799 (1.2) | 118 (0.4) | (0.3) |  |  |  |  |  |  |
|  |  |  |  |  |  |  |  |  |  |
| Race/ethnicity |  |  |  |  |  |  |  |  |  |
| Non-Hispanic White | 1,349,096 (46.9) | 11,990 (41.5) | (0.9) | 567,614 (48.2) | 4,900 (44.4) | (0.9) | 317,386 (53.0) | 2,473 (46.1) | (0.8) |
| Non-Hispanic Black | 407,161 (14.2) | 6,070 (21.0) | (1.5) | 161,373 (13.7) | 1,993 (18.1) | (1.2) | 72,761 (12.2) | 941 (17.5) | (1.3) |
| Hispanic | 673,835 (23.4) | 6,834 (23.6) | (1.0) | 258,387 (21.9) | 2,425 (22.0) | (0.9) | 116,056 (19.4) | 1,153 (21.5) | (1.0) |
| Other | 414,672 (14.4) | 3,970 (13.7) | (1.0) | 189,769 (16.1) | 1,696 (15.4) | (0.9) | 91,944 (15.4) | 795 (14.8) | (0.9) |
| Missing | 29,907 (1.0) | 49 (0.2) | (0.2) | 1,086 (0.1) | 18 (0.2) | (1.7) | 349 (0.1) | 8 (0.1) | (2.3) |
|  |  |  |  |  |  |  |  |  |  |
| Education (years) |  |  |  |  |  |  |  |  |  |
| <8 | 124,193 (4.3) | 1,266 (4.4) | (1.0) | 34,400 (2.9) | 333 (3.0) | (1.0) | 12,950 (2.2) | 142 (2.6) | (1.1) |
| 9-12 | 1,027,058 (35.7) | 12,079 (41.8) | (1.2) | 399,896 (33.9) | 4,086 (37.0) | (1.0) | 185,323 (31.0) | 1,924 (35.8) | (1.0) |
| 13-16 | 1,197,520 (41.7) | 11,294 (39.1) | (0.9) | 511,146 (43.4) | 4,763 (43.2) | (0.9) | 286,613 (47.9) | 2,385 (44.4) | (0.8) |
| ≥17 | 410,625 (14.3) | 3,558 (12.3) | (0.9) | 186,693 (15.8) | 1,623 (14.7) | (0.9) | 107,653 (18.0) | 848 (15.8) | (0.8) |
| Missing | 115,275 (4.0) | 716 (2.5) | 716 (0.6) | 46,094 (3.9) | 227 (2.1) | (0.5) | 5,957 (1.0) | 71 (1.3) | (1.2) |
|  |  |  |  |  |  |  |  |  |  |
| Marital status |  |  |  |  |  |  |  |  |  |
| Single | 902,035 (31.4) | 11,379 (39.4) | (1.3) | 428,332 (36.4) | 4,523 (41.0) | (1.1) | 151,012 (25.2) | 1,798 (33.5) | (1.2) |
| Married | 1,921,635 (66.8) | 17,480 (60.5) | (0.9) | 737,460 (62.6) | 6,503 (58.9) | (0.9) | 446,610 (74.6) | 3,568 (66.4) | (0.8) |
| Missing | 51,001 (1.8) | 54 (0.2) | (0.1) | 12,437 (1.1) | 6 (0.1) | (0.0) | 874 (0.1) | 4 (0.1) | (0.5) |
|  |  |  |  |  |  |  |  |  |  |
| Smoking during pregnancy | |  |  |  |  |  |  |  |  |
| Non-smoker | 2,573,915 (89.5) | 24,885 (86.1) | (1.0) | 1,060,932 (90.0) | 9,921 (89.9) | (0.9) | 555,584 (92.8) | 4,759 (88.6) | (0.9) |
| Smoker | 225,851 (7.9) | 4,007 (13.9) | (1.8) | 86,910 (7.4) | 1,105 (10.0) | (1.3) | 42,029 (7.0) | 610 (11.4) | (1.5) |
| Missing | 74,905 (2.6) | 21 (0.1) | (0.0) | 30,387 (2.6) | 6 (0.1) | (0.0) | 883 (0.1) | 1 (0.0) | (0.1) |
|  |  |  |  |  |  |  |  |  |  |
| Insurance |  |  |  |  |  |  |  |  |  |
| Medicare | 7,615 (0.3) | 115 (0.4) | (1.5) | 2,784 (0.2) | 37 (0.3) | (1.3) | 1,754 (0.3) | 24 (0.4) | (1.4) |
| Medicaid | 449,952 (15.7) | 5,296 (18.3) | (1.2) | 173,927 (14.8) | 1,792 (16.2) | (1.0) | 66,257 (11.1) | 727 (13.5) | (1.1) |
| Private | 1,785,582 (62.1) | 17,194 (59.5) | (1.0) | 752,875 (63.9) | 7,043 (63.8) | (0.9) | 435,438 (72.8) | 3,653 (68.0) | (0.8) |
| Self-pay | 140,979 (4.9) | 1,985 (6.9) | (1.4) | 52,804 (4.5) | 633 (5.7) | (1.2) | 19,031 (3.2) | 259 (4.8) | (1.4) |
| Others | 177,165 (6.2) | 2,187 (7.6) | (1.2) | 68,341 (5.8) | 743 (6.7) | (1.1) | 38,740 (6.5) | 431 (8.0) | (1.1) |
| Missing | 313,378 (10.9) | 2,136 (7.4) | (0.7) | 127,498 (10.8) | 784 (7.1) | (0.6) | 37,276 (6.2) | 276 (5.1) | (0.7) |
|  |  |  |  |  |  |  |  |  |  |
| Hypertension |  |  |  |  |  |  |  |  |  |
| Normotensive | 2,439,904 (84.9) | 23,300 (80.6) | (1.0) | 977,798 (83.0) | 8,596 (77.9) | (0.9) | 543,021 (90.7) | 4,590 (85.5) | (0.8) |
| Chronic hypertension | 32,035 (1.1) | 580 (2.0) | (1.8) | 10,735 (0.9) | 173 (1.6) | (1.6) | 7,172 (1.2) | 106 (2.0) | (1.5) |
| Gestational hypertension | 100,829 (3.5) | 1,353 (4.7) | (1.3) | 53,084 (4.5) | 558 (5.1) | (1.1) | 17,957 (3.0) | 215 (4.0) | (1.2) |
| Mild Preeclampsia | 47,665 (1.7) | 1,008 (3.5) | (2.1) | 29,949 (2.5) | 548 (5.0) | (1.8) | 6,215 (1.0) | 134 (2.5) | (2.2) |
| Severe Preeclampsia | 25,406 (0.9) | 1,131 (3.9) | (4.5) | 15,683 (1.3) | 626 (5.7) | (4.0) | 3,304 (0.6) | 154 (2.9) | (4.7) |
| Superimposed preeclampsia | 3,770 (0.1) | 157 (0.5) | (4.2) | 2,168 (0.2) | 85 (0.8) | (3.9) | 437 (0.1) | 11 (0.2) | (2.5) |
| Eclampsia | 9,845 (0.3) | 314 (1.1) | (3.2) | 4,125 (0.4) | 95 (0.9) | (2.3) | 1,800 (0.3) | 56 (1.0) | (3.1) |
| Missing | 215,217 (7.5) | 1,070 (3.7) | (0.5) | 84,687 (7.2) | 351 (3.2) | (0.4) | 18,590 (3.1) | 104 (1.9) | (0.6) |
|  |  |  |  |  |  |  |  |  |  |
| Diabetes |  |  |  |  |  |  |  |  |  |
| Non-diabetic | 2,685,968 (93.4) | 27,050 (93.6) | (1.0) | 1,115,506 (94.7) | 10,416 (94.4) | (0.9) | 564,269 (94.3) | 5,054 (94.1) | (0.9) |
| Pre-pregnancy | 31,358 (1.1) | 408 (1.4) | (1.3) | 10,827 (0.9) | 140 (1.3) | (1.3) | 6,960 (1.2) | 68 (1.3) | (1.0) |
| Gestational | 119,543 (4.2) | 1,159 (4.0) | (1.0) | 45,471 (3.9) | 394 (3.6) | (0.9) | 26,509 (4.4) | 236 (4.4) | (0.9) |
| Missing | 37,802 (1.3) | 296 (1.0) | (0.8) | 6,425 (0.5) | 82 (0.7) | (1.3) | 758 (0.1) | 12 (0.2) | (1.6) |
|  |  |  |  |  |  |  |  |  |  |
| Inter-pregnancy interval (mon) |  |  |  |  |  |  |  |  |  |
| <6 |  |  |  |  |  |  | 15 (0.0) | 0 (0.0) | (0.0) |
| 6-11 |  |  |  |  |  |  | 5,560 (0.9) | 166 (3.1) | (3.0) |
| 12-23 |  |  |  |  |  |  | 145,886 (24.4) | 1,305 (24.3) | (0.9) |
| 24-35 |  |  |  |  |  |  | 172,645 (28.8) | 1,250 (23.3) | (0.7) |
| 36-47 |  |  |  |  |  |  | 104,521 (17.5) | 865 (16.1) | (0.8) |
| 48-59 |  |  |  |  |  |  | 60,616 (10.1) | 519 (9.7) | (0.9) |
| 60-71 |  |  |  |  |  |  | 36,980 (6.2) | 359 (6.7) | (1.0) |
| 72-83 |  |  |  |  |  |  | 23,202 (3.9) | 274 (5.1) | (1.2) |
| ≥84 |  |  |  |  |  |  | 49,071 (8.2) | 632 (11.8) | (1.3) |
|  |  |  |  |  |  |  |  |  |  |

**Table S3**

**Unadjusted associations between placental abruption and cardiovascular events (mortality and incident non-fatal complications) among the first singleton delivery only and time-varying placental abruption in the first and second singleton deliveries using multi-state models:
*Placental Abruption and Cardiovascular Event Risk (PACER)*, 1993-2020**

|  | **Abruption in the first delivery (Parity 1)** | **First two deliveries (Parity 1 and 2)** | | |
| --- | --- | --- | --- | --- |
|  |  | **Abruption in first delivery only** | **Abruption in second delivery only** | **Abruption in both deliveries** |
|  |  |  |  |  |
| **Cardiovascular disease events** |  |  |  |  |
| Delivery to non-fatal CVD hospitalisation | 1.39 (1.21-1.59) | 1.13 (0.99-1.29) | 1.34 (1.18-1.52) | 1.53 (1.27-1.84) |
| Delivery to all-cause death | 1.89 (1.38-2.58) | 1.07 (0.69-1.64) | 1.69 (1.18-2.42) | 4.52 (1.51-13.53) |
| Non-fatal CVD hospitalisation to all-cause mortality | 0.74 (0.48-1.48) | 0.74 (0.48-1.48) | 0.69 (0.41-0.88) | 1.12 (0.34-3.68) |
|  |  |  |  |  |
| **Heart disease events** |  |  |  |  |
| Delivery to non-fatal heart disease hospitalisation | 1.40 (1.21-1.61) | 1.14 (0.99-1.32) | 1.31 (1.15-1.50) | 1.58 (1.29-1.94) |
| Delivery to all-cause death | 1.85 (1.37-2.51) | 1.10 (0.74-1.65) | 1.66 (1.18-2.34) | 4.09 (1.37-12.26) |
| Non-fatal heart disease hospitalisation to all-cause mortality | 0.74 (0.47-1.17) | 0.74 (0.47-1.17) | 0.61 (0.41-0.92) | 1.20 (0.35-4.03) |
|  |  |  |  |  |
| **Stroke events** |  |  |  |  |
| Delivery to non-fatal stroke hospitalisation | 1.50 (1.10-2.05) | 1.23 (0.87-1.72) | 1.53 (1.08-2.16) | – |
| Delivery to all-cause death | 1.70 (1.29-2.23) | 1.34 (0.97-1.84) | 1.76 (1.35-2.31) | 2.66 (4.65-8.11) |
| Non-fatal stroke hospitalisation to all-cause mortality | 1.12 (0.48-2.64) | 1.12 (0.48-2.64) | 0.69 (0.26-1.84) | – |
|  |  |  |  |  |

Table entries denote the unadjusted hazard ratio and 95% confidence interval based on the multi-state Cox proportional hazards model.

The Cox proportional hazards model was weighted by inverse probability weights to account for missing linkage at first delivery.

All hazard ratios were estimated from results pooled from models based on 25 multiply imputed datasets, with no abruption in the first delivery (parity 1 analysis) or no abruption in the first and second delivery (parity 1 and 2 analysis) as the reference.

**Table S4**

**Rates for placental abruption and cardiovascular events (mortality and incident non-fatal complications) among the first singleton delivery only and time-varying placental abruption in the first and second singleton deliveries:
*Placental Abruption and Cardiovascular Event Risk (PACER)*, 1993-2020**

|  | **First delivery only (Parity 1)** | | **First two deliveries (Parity 1 and 2)** | | | |
| --- | --- | --- | --- | --- | --- | --- |
|  | **No Abruption (Rate)^a^** | **Abruption (Rate)** | **No Abruption (Rate)** | **Abruption at first delivery only (Rate)** | **Abruption at second delivery only (Rate)** | **Abruption at both deliveries (Rate)** |
|  |  |  |  |  |  |  |
| **Total Person-years** | 7,404,614 | 79,735 | 9,502,880 | 75,912 | 83,415 | 2,532 |
|  |  |  |  |  |  |  |
| **Mortality** |  |  |  |  |  |  |
| All-cause | 4,902 (66) | 82 (103) | 4,038 (42) | 46 (61) | 68 (82) |  |
| Cardiovascular disease | 443 (6) | 13 (16) | 331 (3) |  | 6 (7) |  |
| Heart disease | 327 (4) | 8 (10) | 231 (2) |  |  |  |
| Stroke | 116 (2) |  | 100 (10) |  |  |  |
|  |  |  |  |  |  |  |
| **Non-fatal cardiovascular disease (any)** | 19,244 (260) | 268 (336) | 29,628 (312) | 281 (370) | 363 (435) | 13 (513) |
| Heart disease (any) | 16,868 (228) | 235 (295) | 26,132 (275) | 248 (327) | 322 (386) | 12 (474) |
| Ischemic heart disease | 3,694 (50) | 76 (95) | 4,354 (46) | 32 (42) | 62 (74) |  |
| Atherosclerotic heart disease | 1,214 (16) | 12 (15) | 1,734 (18) | 22 (29) | 19 (23) |  |
| Acute myocardial infarction | 970 (13) | 23 (29) | 1,132 (12) | 6 (8) | 18 (22) |  |
| Hypertensive heart disease | 717 (10) | 6 (8) | 808 (9) | 8 (11) | 11 (13) |  |
| Heart failure | 1,663 (22) | 23 (29) | 1,678 (18) | 21 (28) | 21 (25) |  |
| Cardiomyopathy | 865 (12) | 11 (14) | 974 (10) | 11 (14) | 14 (17) |  |
| Cardiac arrhythmias | 10,455 (141) | 134 (168) | 18,347 (193) | 170 (224) | 212 (254) | 7 (276) |
|  |  |  |  |  |  |  |
| Stroke (any) | 2,697 (36) | 41 (51) | 3,847 (40) | 42 (55) | 46 (55) |  |
| Hemorrhagic stroke | 639 (9) | 6 (8) | 818 (9) | 10 (13) | 15 (18) |  |
| Ischemic stroke | 2,055 (28) | 36 (45) | 2,995 (32) | 30 (40) | 32 (38) |  |

^a^Rates are expressed per 100,000 person-years.

**Table S5**

**Unadjusted associations between placental abruption and cardiovascular events (mortality and incident non-fatal complications) among the first singleton delivery only and time-varying placental abruption in the first and second singleton deliveries based on traditional Cox proportional hazards regression models: *Placental Abruption and Cardiovascular Event Risk (PACER)*, 1993-2020**

|  | **Abruption at first delivery (Parity 1)** | **First two deliveries (Parity 1 and 2)** | | |
| --- | --- | --- | --- | --- |
|  |  | **Abruption at first delivery only** | **Abruption at second delivery only** | **Abruption at both deliveries** |
|  |  |  |  |  |
| **Mortality** |  |  |  |  |
| All-cause | 1.45 (1.17-1.81) | 1.41 (1.04-1.89) | 1.72 (1.34-2.21) | 4.22 (1.67-10.66) |
| Cardiovascular disease | 2.22 (1.25-3.92) | – | 1.84 (0.80-4.22) | – |
| Heart disease | 1.85 (0.89-3.86) | – | 2.11 (0.83-5.35) | – |
| Stroke | 3.15 (1.27-7.80) | 2.28 (0.52-9.92) | – | – |
|  |  |  |  |  |
| **Non-fatal cardiovascular disease (any)** | 1.21 (1.07-1.37) | 1.16 (1.03-1.31) | 1.39 (1.25-1.54) | 1.52 (0.86-2.29) |
| Heart disease (any) | 1.23 (1.08-1.40) | 1.17 (1.03-1.32) | 1.39 (1.24-1.55) | 1.60 (0.86-2.88) |
| Ischemic heart disease | 1.82 (1.46-2.28) | 0.83 (0.58-1.20) | 1.62 (1.27-2.07) | – |
| Atherosclerotic heart disease | 0.91 (0.53-1.59) | 1.65 (1.08-2.50) | 1.38 (0.87-2.06) | 3.90 (0.91-16.75) |
| Acute myocardial infarction | 2.29 (1.55-3.37) | 0.60 (0.25-1.38) | 1.79 (1.15-2.81) | 2.97 (0.38-23.35) |
| Hypertensive heart disease | 0.69 (0.31-1.51) | 1.27 (0.65-2.49) | 1.16 (0.61-2.23) | - |
| Heart failure | 1.14 (0.76-1.73) | 1.41 (0.90-2.22) | 1.33 (0.87-2.05) | 2.18 (0.31-15.42) |
| Cardiomyopathy | 1.07 (0.59-1.96) | 1.10 (0.56-2.15) | 1.40 (0.80-2.43) | 3.54 (0.47-26.68) |
| Cardiac arrhythmias | 1.12 (0.94-1.33) | 1.15 (0.98-1.34) | 1.32 (1.15-1.51) | 1.31 (0.610-2.85) |
|  |  |  |  |  |
| Stroke (any) | 1.23 (0.89-1.70) | 1.31 (0.96-1.79) | 1.41 (1.06-1.86) | – |
| Hemorrhagic stroke | 0.85 (0.39-1.91) | 1.62 (0.89-2.95) | 2.08 (1.27-3.41) | – |
| Ischemic stroke | 1.39 (0.98-1.95) | 1.15 (0.79-1.68) | 1.29 (0.93-1.80) | – |
|  |  |  |  |  |

The Cox proportional hazards model was weighted by inverse probability weights to account for missing linkage at first delivery.

All hazard ratios were estimated from results pooled from models based on 25 multiply imputed datasets, with no abruption in the first delivery (parity 1 analysis) or no abruption in the first and second delivery (parity 1 and 2 analysis) as the reference.

**Table S6**

**Adjusted associations between placental abruption and cardiovascular events (mortality and incident non-fatal complications) among the first singleton delivery only and time-varying placental abruption in the first and second singleton deliveries based on traditional Cox proportional hazards regression models: *Placental Abruption and Cardiovascular Event Risk (PACER)*, 1993-2020**

|  | **Abruption in first delivery (Parity 1)** | **First two deliveries (Parity 1 and 2)** | | |
| --- | --- | --- | --- | --- |
|  |  | **Abruption at first delivery only^a^** | **Abruption at second delivery only^b^** | **Abruption at both deliveries^b^** |
|  |  |  |  |  |
| **Mortality** |  |  |  |  |
| All-cause | 1.21 (0.97-1.51) | 1.21 (0.91-1.64) | 1.42 (1.11-1.82) | 2.99 (1.19-7.52) |
| Cardiovascular disease | 1.68 (0.95-2.99) | – | 1.39 (0.60-3.20) | – |
| Heart disease | 1.41 (0.67-2.93) | – | 1.62 (0.64-4.12) | – |
| Stroke | 2.34 (0.94-5.85) | 1.57 (0.36-6.86) | – | – |
|  |  |  |  |  |
| **Non-fatal cardiovascular disease (any)** | 1.05 (0.93-1.19) | 1.07 (0.85-1.21) | 1.23 (1.11-1.36) | 1.16 (0.66-2.05) |
| Heart disease (any) | 1.07 (0.94-1.22) | 1.07 (0.94-1.22) | 1.23 (1.10-1.37) | 1.22 (0.68-2.21) |
| Ischemic heart disease | 1.53 (1.22-1.91) | 0.75 (0.52-1.08) | 1.37 (1.07-1.76) | – |
| Atherosclerotic heart disease | 0.77 (0.44-1.34) | 1.55 (1.02-2.36) | 1.19 (0.77-1.83) | 3.12 (0.73-13.43) |
| Acute myocardial infarction | 1.89 (1.28-2.79) | 0.53 (0.23-1.22) | 1.48 (0.94-2.34) | 2.02 (0.26-15.92) |
| Hypertensive heart disease | 0.52 (0.24-1.15) | 1.03 (0.53-2.01) | 0.77 (0.40-1.48) | – |
| Heart failure | 0.86 (0.57-1.31) | 1.17 (0.74-1.86) | 0.95 (0.62-1.47) | 1.02 (0.14-7.22) |
| Cardiomyopathy | 0.85 (0.46-1.55) | 0.98 (0.49-1.96) | 1.11 (0.64-1.94) | 2.05 (0.27-15.67) |
| Cardiac arrhythmias | 1.02 (0.85-1.21) | 1.07 (0.92-1.26) | 1.22 (1.06-1.39) | 1.11 (0.51-2.41) |
|  |  |  |  |  |
| Stroke (any) | 1.04 (0.75-1.43) | 1.18 (0.86-1.61) | 1.21 (0.92-1.61) | – |
| Hemorrhagic stroke | 0.72 (0.32-1.61) | 1.44 (0.79-2.62) | 1.71 (1.04-2.80) | – |
| Ischemic stroke | 1.16 (0.82-1.64) | 1.04 (0.71-1.51) | 1.13 (0.81-1.56) | – |
|  |  |  |  |  |

The Cox proportional hazards model was weighted by inverse probability weights to account for missing linkage at first delivery, and the results were pooled from 25 multiply imputed datasets.

All hazard ratios were estimated from results pooled from models based on 25 multiply imputed datasets, with no abruption in the first delivery (parity 1 analysis) or no abruption in the first and second delivery (parity 1 and 2 analysis) as the reference.

1. Hazard ratios were adjusted for confounding effects of the mother’s age, mother’s race (non-Hispanic Black, Hispanic, non-Hispanic White, and other races (Indian [North American, Central American, South American, Eskimo, and Aleut], Chinese, Japanese, Hawaiian, Filipino, Other Asian [Pakistani, Bangladeshi, Cambodian, Thai], Unknown, Asian Indian, Korean, Samoan, Vietnamese, Guamian), marital status, mother’s education, insurance, smoking status, hypertensive disorders of pregnancy (chronic hypertension, gestational hypertension, preeclampsia, and eclampsia), pregestational diabetes, and year of delivery of the first pregnancy.
2. Hazard ratios were adjusted for confounding effects of the mother’s age, mother’s race (non-Hispanic Black, Hispanic, non-Hispanic White, and other races (Indian [North American, Central American, South American, Eskimo, and Aleut], Chinese, Japanese, Hawaiian, Filipino, Other Asian [Pakistani, Bangladeshi, Cambodian, Thai], Unknown, Asian Indian, Korean, Samoan, Vietnamese, Guamian), marital status, mother’s education, insurance, smoking status, hypertensive disorders of pregnancy (chronic hypertension, gestational hypertension, preeclampsia, and eclampsia), pregestational diabetes, interpregnancy interval, and year of delivery of the first and second pregnancies.
